# Urachal Tumor: A Case Report of an Extremely Rare Carcinoma

**DOI:** 10.1155/2017/1942595

**Published:** 2017-02-22

**Authors:** José Palla Garcia, Rita Sampaio, Carlos Peixoto

**Affiliations:** Department of Pathology, Pathological Anatomy Service, Centro Hospitalar do Porto, Largo Professor Abel Salazar, 4099-003 Porto, Portugal

## Abstract

The urachus is a tubular structure that connects the bladder to the allantois in the embryonic development, involuting after the third trimester. The urachus carcinoma is an extremely rare tumor that accounts for <1% of all bladder cancers. We report a case of a 46-year-old woman, with no past medical history, complaining of hematuria with 6-month duration and a physical exam and an abdominal computed topographic scan revealing an exophytic mass of 6.8 cm longer axis that grew depending on the anterior bladder wall, invading the anterior abdominal wall. Cystoscopy detected mucosal erosion. The biopsy showed structures of adenocarcinoma of enteric type. The surgical specimen showed urachus adenocarcinoma of enteric type with stage IVA in the Sheldon system and stage III in the Mayo system. This case has a 3-year follow-up without disease recurrence.

## 1. Introduction

The urachus is a tubular structure that connects the bladder to the allantois in the embryonic development, involuting after the third trimester, into a fibromuscular tract or closed canal between the dome of urinary bladder and the umbilicus. Urachal remnant may persist in approximately 32% of adults [1], consisting of a tubular or cystic structure lined by epithelium, surrounded by connective tissue and musculature, in the complete form.

The urachus carcinoma accounts for <1% of all bladder cancers [2, 3]. It is a malignant epithelial neoplasm arising from urachal remnants. Ninety percent of them are adenocarcinomas [4], believed to evolve from intestinal metaplasia of the epithelial component [1], accounting for 10% of the bladders adenocarcinoma [5]. Nonglandular neoplasms can be urothelial, squamous cells, neuroendocrine, and mixed type [6].

To date, because of its rarity, there is some inconsistency and no consensus in the literature about the nomenclature, the diagnostic criteria, the staging system to use, and the best therapeutic options.

We report a case of 46-year-old woman with an urachal carcinoma and do a brief literature review about this extremely rare entity.

## 2. Case Report

**Figure 1:**
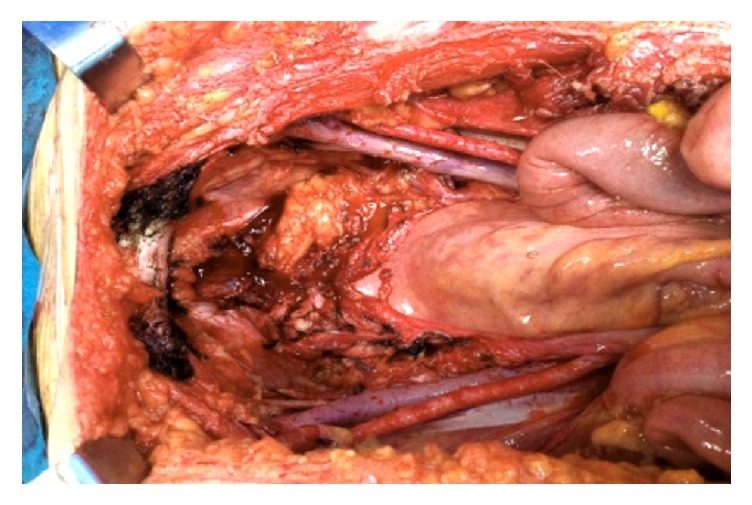
Intraoperative view.

A 46-year-old woman with no past medical history was referred to our hospital with complaints of hematuria with 6-month duration. No other complaint was reported. Physical exam and an abdominal computed topographic (CT) scan revealed an exophytic mass with 6.8 cm longer axis that grew depending on the anterior bladder wall, invading the anterior abdominal wall. Cystoscopy detected mucosal erosion, and a vesical biopsy was performed showing adenocarcinoma structures of enteric type in the bladder wall, partially covered by urothelium with reactive changes. A thoracic CT scan and a virtual colonoscopy were performed showing no obvious sites of distant metastases, or primary disease of the bowel. Based on these findings, a decision was made to operate. The surgical approach adopted was a pelvic exenteration with anterior resection of the abdominal rectum and navel, with pelvic lymphadenectomy and ureteroileostomy of Bricker (Figure 1).

**Figure 2:**
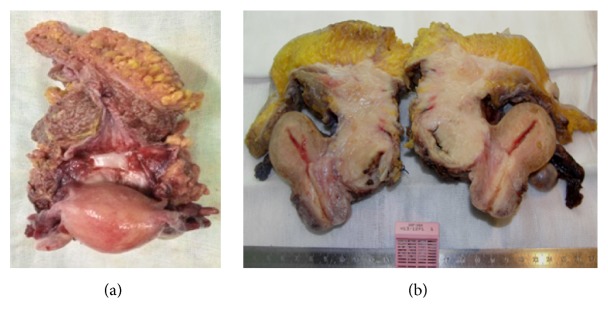
Macroscopic aspects: (a) superior view; (b) sagittal section.

The bladder, uterus, ovaries, navel, subcutaneous tissue, and rectus abdominis muscle composed the complex surgical piece. The bladder with 4 cm internal diameter had a thickened wall in its upper half (3.5 cm thickness) due to diffuse infiltration by whitish and compact tumor with 7.5 cm greatest diameter, centered between the bladder and abdominal wall (which was infiltrated to the level of the subcutaneous tissue). Uterus and ovaries were unchanged (Figure 2).

Samples were fixed in 10% neutral-buffered formalin and embedded in paraffin. Paraffin sections were stained with standard routine stains. Immunostains were performed with avid-biotin-peroxidase method.

**Figure 3:**
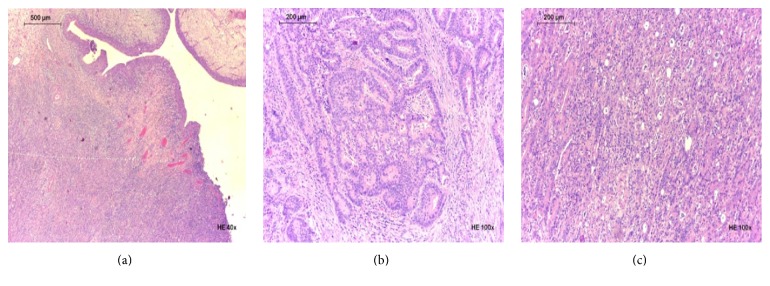
Histologic features of the tumor: (a) urothelium surface; (b) glandular differentiation; (c) areas of lesser differentiation.

**Figure 4:**
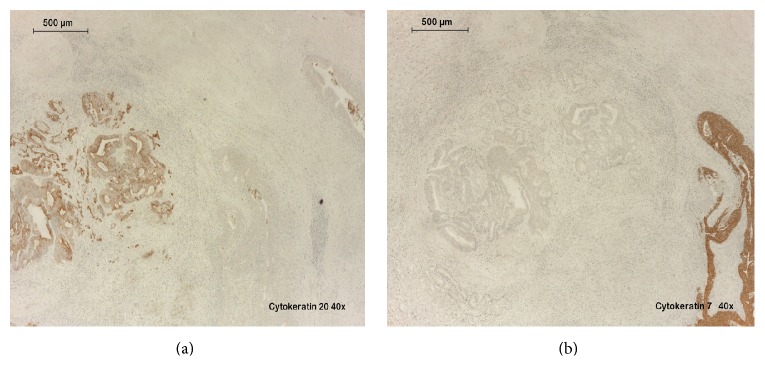
Immunostains: (a) CK20 positive staining in the tumor and negative staining in the overlying urothelium; (b) CK7 negative staining in the tumor and positive staining in the urothelium.

**Figure 5:**
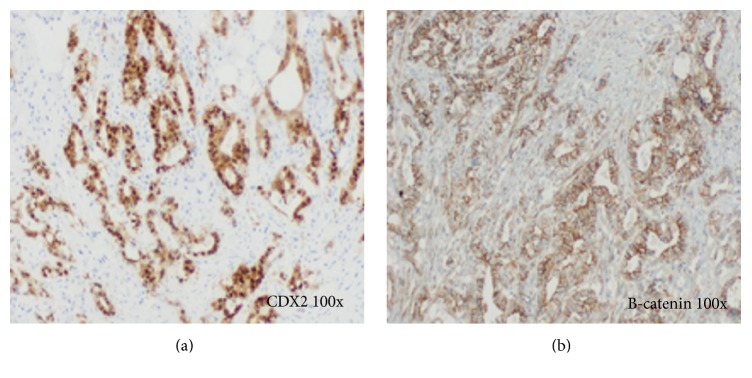
Immunostains: (a) CDX2 positive staining in the tumor; (b) *β*-catenin negative nuclear staining in the majority of the tumor.

The histology revealed an adenocarcinoma of enteric type with poorly differentiated areas and areas with focal mucinous differentiation (Figure 3). The tumor was accompanied by intense inflammatory and desmoplastic reaction. The surgical retropubic plan intersected neoplastic tissue. All the remaining surgical margins were free. Metastasis to a single regional lymph node was identified. The immunostains revealed cytokeratin 20 (CK20) and CDX2 expression, absence of expression for cytokeratin 7 (CK7) and cytokeratin 34*β*E12 (34*β*E12), and only focal nuclear staining for *β*-catenin (Figures 4 and 5).

The patient underwent then 12 cycles of chemotherapy with FOLFOX (5-FU, leucovorin, and oxaliplatin) and pelvic radiotherapy.

The patient has a 3-year clinical and imagiologic follow-up, with no evidence of disease recurrence.

## 3. Discussion

Urachus carcinoma is a rare entity, with quite limited published studies. Hue and Jacquin published the first case report in 1863. In the 1950s Wheeler and Hill proposed the initially accepted diagnostic criteria for the urachus carcinoma. The criteria have undergone modifications and are still controversial; even so, most investigators accepted the proposed criteria by Sheldon et al. [7] and Mostofi et al. [8] that were (a) tumor in the dome of the bladder, (b) absence of cystitis cystica and cystitis glandularis, (c) predominant invasion of the muscularis or deeper tissues with a sharp demarcation between the tumor and surface bladder urothelium that is free of glandular or polypoid proliferation, (d) presence of urachal remnants within the tumor, (e) extension of tumor into the bladder wall with involvement of the space of Retzius, anterior abdominal wall, or umbilicus, and (f) no evidence of a primary neoplasm elsewhere. However, these criteria were considered somewhat restrictive by some studies [9–11]. A new somewhat broader set of criteria adapted from Gopalan et al. was published in the 2016 World Health Organization (WHO) blue book for the diagnosis of urachal adenocarcinoma which are (a) location of the tumor in the bladder dome and/or anterior wall, (b) epicenter of carcinoma in the bladder wall, (c) absence of widespread cystitis cystica and/or cystitis glandularis beyond the dome and anterior wall, and (d) absence of a known primary tumor elsewhere.

The case presented here illustrated the following features: the tumor was located in the anterior wall and in the bladder dome, the epicenter of the tumor was in the bladder wall, there was absence of cystitis cystica or cystitis glandularis, and the investigations carried out did not reveal any primary tumor elsewhere, thereby fulfilling all the WHO criteria for the diagnosis of urachal adenocarcinoma. It showed also a sharp demarcation between the tumor and surface bladder urothelium that was free of glandular and polypoid proliferation. And finally the presence of urachal remnant explained by the tumor extent was not documented. This feature is helpful for the diagnosis but its absence does not preclude the urachal origin.

It was a glandular tumor, with enteric differentiation, being considered an enteric adenocarcinoma. The generally accepted histological subtyping of urachal adenocarcinoma is of enteric, mucinous, signet ring cell, mixed type or not otherwise specified. Most of the adenocarcinomas are of mucinous type [1]. According to same authors, there is no association between prognosis and tumor type [11].

Immunohistochemically (IC), the adenocarcinoma of the present case, expressed CK20 and CDX2, was negative for CK7 and 34*β*E12 and presents only very focal nuclear staining for *β*-catenin. These IC results support the diagnosis of urachal origin. The urachal adenocarcinoma is generally positive for CK20 and CDX2, CK7 in 60% of the cases, and 34*β*E12 in 66% but only very focally [1, 11]; nuclear staining with *β*-catenin occurs in 6% [1], normally showing only cytomembranous staining. The diffuse nuclear staining of *β*-catenin and the absence of expression of CK7 favor colonic origin. The gastrointestinal tract markers claudin-18 and Reg IV can also be expressed by the urachal adenocarcinomas [1].

The molecular alterations reported in 40% of urachal adenocarcinomas are microsatellite instability and mutations of KRAS at codon 12 [6]. The patients with the KRAS mutations have a better overall survival [6]. No other molecular alterations were reported, like BRAF mutations, EGFR mutations, or ALK rearrangements.

The differential diagnosis of this case, like the majority of urachal adenocarcinomas, is between adenocarcinomas of two different origins, primary vesical or metastatic colon tumor. For the exclusion of vesical origin the gross features, the epicenter of the tumor in the bladder wall, a sharp demarcation between the tumor and the surface bladder urothelium, and the absence of carcinoma in situ or extensive glandular metaplasia of the adjacent urothelium must be taken into account. For the exclusion of the colonic origin, a clinical exploration and the results of immunostains are very important.

There are several staging systems for urachal carcinomas. The most widely accepted one is the staging proposed by Sheldon et al. (Table 1) and the Mayo system (Table 2), proposed by Ashley et al., but their relevance still needs validation by larger series.

Most urachal carcinomas are diagnosed in advance stages [9], being associated with a poor prognosis. The overall outcome falls around 45% to 50%, 5-year survival [2, 3, 5, 12]. In comparison with bladder urothelial carcinoma, in similar stages, urachal carcinoma has a better survival rate [9]. There are some independent predictor factors, considered by some studies to influence the outcome [2, 12, 13], that are tumor spread outside of bladder to adjacent organs and/or abdominal wall, the presence of metastasis, and residual disease.

Our case was staged by Sheldon system as stage IVA and stage III by Mayo system. This case despite advance stage of the disease, lymph node metastasis, and the presence of neoplastic structures in surgical margin, has a 3-year clinical and imagiologic follow-up without disease recurrence.

The recommended treatment for nonmetastatic cases is surgery. Partial or radical cystectomy has similar oncologic results [4]. En bloc resection with complete removal of urachal remnant and the umbilicus should be the surgery performed for prolonged survival [4]. The effective role of neoadjuvant or adjuvant chemotherapy is still to be proven [1]; however there are some reports of metastatic cases, with response to FOLFOX chemotherapeutic regimen [1]. Radiotherapy was performed in our case because of the positive surgical margin.

## 4. Conclusion

In summary, we present a case of urachal adenocarcinoma, of enteric type, with advance stage disease, with some negative predictor factors like lymph node metastasis, and with positive surgical margin. The patient underwent surgical resection of the tumor, chemotherapy, and radiotherapy and has a 3-year follow-up free of recurrence of disease. In the literature review there is a new set of criteria adapted from Gopalan et al. and published in the 2016 WHO blue book, and the most accepted staging systems are still the Sheldon and Mayo systems. Relatively to the outcome there are some studies reporting better survival rates of urachal adenocarcinoma comparing with the urothelial carcinoma.

## Figures and Tables

**Table 1:** Sheldon staging system.

I	No invasion beyond the urachal mucosa
II	Invasion confined to the urachus
III	Local extension into
IIIA	Bladder
IIIB	Abdominal wall
IIIC	Peritoneum
IIID	Viscera other than the bladder
IV	Metastasis to
IVA	Regional lymph nodes
IVB	Distant sites

**Table 2:** Mayo staging system.

I	Confined to the urachus and/or bladder
II	Extension beyond the muscular layer of the urachus and/or bladder
III	Infiltration to the regional lymph nodes
IV	Infiltration to nonregional lymph nodes or other distant sites
